# Comparative Evaluation of Shear Bond Strength of Aesthetic Orthodontic Brackets Bonded to Aged Composite Restorative Resin Materials

**DOI:** 10.3390/polym17050621

**Published:** 2025-02-26

**Authors:** Mohammed E. Sayed

**Affiliations:** Department of Prosthetic Dental Sciences, College of Dentistry, Jazan University, Jazan 45142, Saudi Arabia; mesayed@jazanu.edu.sa or drsayed203@gmail.com; Tel.: +966-506-529-134

**Keywords:** flowable composites, packable composites, ceramic brackets, plastic brackets, orthodontic tooth movement

## Abstract

Patient demands for aesthetic orthodontic brackets (OBs) has increased since orthodontic treatments are of long duration. Clinicians encounter old composite restorations frequently, against which OBs need to be bonded. This study aims to determine the shear bond strength (SBS) of two aesthetic OBs (ceramic and resin) against aged composite resins (flowable and packable) after standard surface treatment. A total of 96 disk-shaped specimens of two aged (A) composite resins [flowable (F) and packable (P)] were divided into eight groups, using ceramic (C) and plastic (P) brackets, out of which four subgroups served as the control [non-aged (N)FC, NPC, NFR, NPR] and four as experimental [AFC, APC, AFR, APR]. Surface treatment included mechanical [air abrasion] and chemical [Assure Plus and Transbond XT]. After 24 h of storage, the specimens were tested for SBS and observed for failure mode using adhesive remnant index scores. Mean values of SBS in each subgroup were analyzed statistically using a one-way analysis of variance test and Tukey post hoc test. All probability ‘*p*’ differences were significant at a value of 0.05 and less. All aged composite resin subgroups had decreased bond strength than controls, with all subgroups bonded with plastic brackets having the least bond strengths that were clinically nonacceptable [≤7 to 10 MPa]. Flowable composites when bonded with either ceramic or plastic brackets had higher strength than packable composites. Ceramic brackets had higher SBS than plastic brackets for both flowable and packable composites. Significant differences in bond strength were observed among subgroups of plastic brackets. Ceramic brackets were associated with a higher residue of adhesives on the composite surface. Aged composite resins exhibit significantly lower SBS than fresh composites, with ceramic brackets and flowable composites producing better bond strength values than plastic brackets and packable composites.

## 1. Introduction

The adult population has been gradually increasing their desire for orthodontic treatment, which has resulted in a manifold increase among dental outpatients. Many of these patients also seek treatment using aesthetic/orthodontic brackets (OBs) rather than conventional stainless steel brackets. With such patient demands, the orthodontists as well as the manufacturers have evolved, introducing new orthodontic brackets. Given that many patients have had their existing teeth restored using a variety of restorative materials such as composite resin, amalgam, and porcelain, orthodontists are more likely to encounter difficulties when attempting to bond orthodontic braces (OBs) to these restorative materials [1,2]. Other dental disciplines, in addition to routine orthodontic patients, seek orthodontic interventions as part of prerestorative mouth preparations, orthodontic root extrusions, and complete occlusal rehabilitations to achieve their respective treatment goals [3]. Intradisciplinary preventive orthodontics in deciduous or mixed dentitions require bonding brackets in genetic dental conditions (for example, amelogenesis/dentinogenesis imperfecta), tooth fracture, and gross decay that require a restoration placement (single crown, temporary bridge) before commencing the tooth movements [4]. Prosthodontic–orthodontic multidisciplinary approaches are also recommended for cases like hypodontia, polydiastema, or implant-less space [2,5]. Orthodontists bond brackets to temporary composite crowns and may also use a composite provisional crown/bridge for an extended period during orthodontic treatment. Provisional composite crowns not only provide temporary protection, stabilization, and function to the patient [6], but also assist orthodontists in determining the final restoration’s aesthetic outcome and exert forces on adjacent teeth [7]. Orthodontic treatment results take time (6 to 30 months) [8]; therefore, the tooth surface or restoration that is utilized for bracket bonding must be able to function for extended periods of time. Shear loads/forces are the most destructive forces that can cause orthodontic bracket debonding, while tensile and torsional forces have a minor impact [9]. Orthodontic treatment requires the application of different forces to accomplish tooth movement. These forces range from 35 to 60 g for extrusion and from 70 to 120 g for tooth translation [10]. The ideal or minimum threshold for an OB bond should be at least in the range of 6 to 8 MPa; however, critics question such threshold values for clinical application since they were established using in vitro tests and do not consider therapeutically relevant variables like pH, temperature, humidity variations, fatigue-related adhesive, and microbiological degradation present in the oral cavity [3,11]. Research shows that in vivo-aged specimens have significantly lower bond strength than in vitro-aged specimens, and other factors like material type, adhesive, storage time, and aging process also significantly influence bracket bond strength [12]. The clinical failure rates of brackets can range from 0.5% to 55.8% [13], with bracket failure rates of mandibular molars [2.7% to 29%] being more common [14], depending on the position, bonding material, and duration. Peak failure can occur after placement or later, after the brackets have endured mouth and treatment challenges. Early bracket loss often results from inadequate technique, fluid contamination, or premature archwire engagement, while excessive mechanical forces can cause extended use loss.

The SBS of OBs has been tested both in vivo [15] and in vitro on natural tooth enamel [16,17,18,19,20,21], various dental porcelain restoration substrates [22,23,24,25], composite restorative resins [1,26,27,28,29,30,31,32,33,34,35], dental amalgam [36,37], provisional crown materials like polymethyl methacrylate [unfilled, milled, three-dimensional printed] [38,39], and composite provisional crowns [1,4,40,41,42,43]. Three-dimensional printing has even found its application in bone tissue scaffolds for repairing large-area irregular bone defects in orthopedic treatment [44]. Orthodontic brackets against which the bond strength has been determined include stainless steel [9,17,22,24,28,29,30,31,38], ceramic [17,18,22,23,24,25,26,27,28,29], and plastic [45,46,47,48,49,50,51,52] types. These studies have constantly observed different SBS on different surfaces, with chemically similar surfaces showing increased bond strength than chemically different surfaces. Compared to enamel, the SBS on various restorative surfaces has been observed to be lower for stainless steel OBs. The literature discusses the effectiveness of different bracket types in improving adherence. Stainless steel brackets, made from alloys like carbon, nickel, molybdenum, titanium, phosphorus, tantalum, selenium, and chromium, offer high stiffness, strength, resilience, biocompatibility, and corrosion resistance [9,28,29,30,31]. However, they require soldering and have a high elastic modulus [9,22]. They bond poorly to ceramic surfaces unless specific surface modifications are made [38]. Ceramic brackets in orthodontics are categorized into polycrystalline and monocrystalline types, offering strength, durability, and aesthetic appeal [22,23,24,25,26,27,28,29]. They are made from alumina and can be enhanced with techniques like mechanical removal and HFA etching but may compromise ceramic integrity [17,18]. Phosphoric acid can improve silane application conditions but does not erode silicate ceramic layers [23,24,25]. There is no accord on the ideal surface conditioning method for optimal bond strength, and their high bond strength to adhesives can cause enamel damage [22,23,24,25,26,27,28,29]. Plastic brackets offer tinting and soft edges but have disadvantages like initial weakness, bonding issues, and issues like tie wing fractures and warping [45,46,47,48,49]. Brackets made of high-quality medical polyurethane or polycarbonate with ceramic or fiberglass reinforcements fix these problems, making them more effective and decreasing torque loss [45,47,51]. Ceramic-reinforced polycarbonate brackets experience the most deformation under torque [45,50]. For clinical use, plastic brackets are more effective when incorporating a metal groove to enhance strength and reduce breakage compared to stainless steel brackets [48,51]. Iwasaki T et al. [53], while examining the mechanical properties of cosmetic orthodontic brackets, including ceramic and plastic ones, observed significant differences in mechanical characteristics between monocrystalline and polycrystalline ceramic brackets, with glass fiber-reinforced plastic brackets showing better performance. Another influential factor affecting the bond strength of various OBs with bonding surfaces is the surface treatment employed [3,9,29,30,35,38,41,42,43]. These methods include mechanical approaches like sandblasting and grinding with diamond or carbide burs, which aim to roughen the surface and enhance bonding areas. Chemical methods, such as etching with phosphoric acid and hydrofluoric acid, are also employed to prepare surfaces for orthodontic bonding. Specific techniques for crown materials include diamond burs, sandblasting, acid etching, and Er:YAG laser systems for porcelain surfaces. For temporary crowns, aluminum oxide particle air blasting, diamond burs, and hydrofluoric acid etching are commonly used. Overall, the choice of surface treatment method is crucial. Airblasting using sand (sandblasting with alumina particles) is a universally accepted standard for surface treatment for all types of OBs [41] on most of the restoration surfaces [14,19,43].

Research on the aging of the bracket–surface bond [3,42] in the oral cavity has shown a significant decrease in SBS between the OB and restorative materials. Another clinically significant aspect that has caught researchers’ attention in recent years is the influence of aged restorations on the SBS of orthodontic brackets [1,2,28,29,30,31]. Bayram M et al. [1] studied surface conditioning [38% phosphoric acid, 9.6% hydrofluoric acid, airborne aluminum trioxide, sodium bicarbonate particle, diamond bur] of metal brackets and aged composite [1000 cycles], with air abrasion showing the highest SBS at 10.29 MPa, although all surface treatments showed clinically acceptable strengths. Blakey R et al. [7] examined the impact of surface modifications on the SBS of OBs [metal and ceramic] bonded to polycarbonate crowns and found sandblasting as the single treatment that significantly affected the bond strength. Della Bona A et al. [28] assessed the SBS of metal and ceramic brackets bonded to aged [23 days in 37 °C deionized water] resin-based composite restorations after various surface treatments (acid etch with 38% H_3_PO_4_ for 20 s, surface roughening with bur, then acid etch). Results showed similar SBS values, with metal brackets showing greater due to mechanical retention. The study concluded sufficient bond strength was produced on aged composites regardless of bracket type. Eslamian L et al. [29], while assessing the SBS of metal OBs to aged nanohybrid composite [500 cycles], found diamond bur roughening significantly improved the SBS to aged composite while at the same time increasing the adhesive remnant index (ARI). Sandblasting, however, was not compared in this study. Farhadifard H et al. [54], in his study on ceramic bracket bond to aged [30 days in 37-degree deionized water] nanohybrid composite restoration, found sandblasting [8.13 MPa] to be more effective than diamond grinding [9.16 MPa]. Tayebi A et al. [34] used sandblasting and diamond bur followed by different primer applications on aged [2000 thermal cycles] bulk fill composite [Filtek Z250] and found that metal brackets showed higher SBS after sandblasting [9.94 to 13.8 MPa] followed by diamond bur [7.57 to 10.8 MPa]. Valizadeh S et al. [31] examined the impact of acid etchant and different adhesives, bur, sandblasting, and Er:YAG laser on the SBS of metal brackets to aged composite (10,000 cycles). In the no-preparation and laser groups, there was no discernible difference in SBS between the adhesives, but sandblasting yielded the greatest SBS across all groups. Seyhan-Cezairli N et al. [55], in his study on the SBS of metal brackets to three different aged (500 cycles between 5 °C and 55 °C) bulk fill composites using a single surface treatment, observed a decline in SBS for all aged composites when compared to the control, indicating that aging actually reduces SBS.

The analysis of the literature shows that these studies have primarily used stainless steel OBs on different types of bulk fill composite resins. Studies investigating the SBS of ceramic and plastic (resin) OBs are missing. Another area of research interest indicates that flowable composites, which have been refined over the last decade, have never been investigated. Danha LS et al. [24], recently studied the SBS between sapphire brackets and aged bulk fill composite resin [5000 cycles], with flowable composite used for bonding the bracket base to the bulk fill composite disks. The results showed significantly lower SBS. Goracci C et al. [21] had previously attempted the same experiment and found similar results. The use of flowable composite therefore has not been investigated, although its clinical use has significantly grown in recent years [24]. Therefore, this study was aimed at determining the SBS of ceramic and plastic brackets to aged [10,000 cycles] flowable and packable composite restorative resins using a standard surface treatment (mechanical and chemical) protocol. The objective of the study is to identify whether old composite restorations should be replaced with fresh ones before bonding ceramic and plastic OBs to them. The study hypothesizes that aged composite restorations will have less SBS than fresh ones, which will also reflect in a lower ARI score. Conversely, the study’s null hypothesis asserts that there will be no differences in SBS between aged and fresh composite restorations for both ceramic and resin brackets. This study emphasizes the importance of assessing the bond strength of aged composite restorations with aesthetic OBs to improve treatment outcomes, especially for composite restorations commonly encountered in clinical practice.

## 2. Materials and Methods

**Figure 1:**
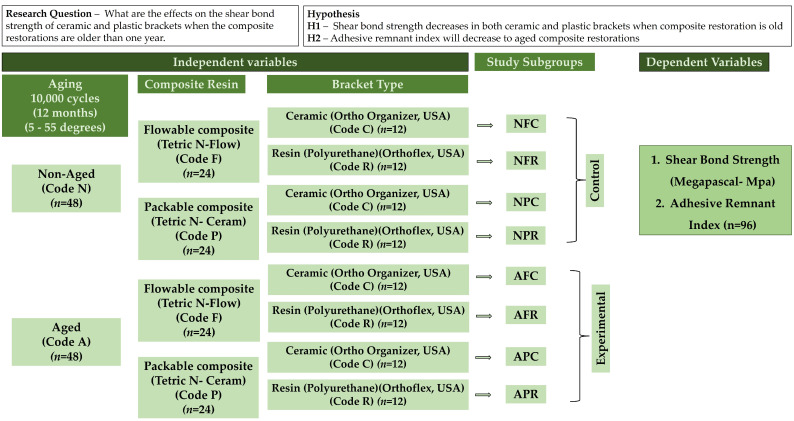
Flowchart showing hypothesis, independent and dependent variables, and various subgroups involved in the study.

*Study Design*: This in vitro research followed a comparative (control/experimental) strategy, wherein the restorative specimens underwent an aging process followed by surface treatment and bracket bonding placement before testing for SBS. The study evaluated four test groups by comparing their results with the respective four control groups. The study sequence is depicted as a flowchart in Figure 1, with clearly defined independent and dependent variables.

*Definitions of Operational Terms* [56]: The term “adhesive failure” was defined as the bond failure that occurred at the interface between two dissimilar materials as a result of forces (tensile or shear). Cohesive failure is described as the failure of a bond inside a dental material as a result of shear or tensile force (American Dental Association specified). SBS referred to the maximum force that an adhesive joint is capable of withstanding before it fails or fractures. Internally induced forces that oppose the sliding of one plane on a neighboring plane are referred to as shear stress. Shear stress may also be thought of as the force that resists a twisting motion as well.

*Size of the Sample*: The configuration of the research project consisted of two primary groups on the basis of aging [aged and non-aged], each of which contained four subgroups depending upon the type of composite resin (flowable and packable). Each subgroup was further divided into two subgroups depending upon the type of aesthetic bracket bonded (ceramic and plastic), leading to the formation of eight different working groups [four control and four experimental]. Based on the total number of subgroups with an effect size of D2 = 0.28, a power assumption of 80%, and a type 1 error rate of 0.05, the total number of samples required was calculated using the formula (N = 2 σ2 × (Z α + Z β) 2/2) on Nquery software (v7.0; Informer Technologies, Los Angeles, CA, USA) [57], which came out to be 96 specimens, with each subgroup having 12 specimens. In case a sample turned out to be defective and not fit for use, two replacement samples for each subgroup were prepared as reserves.

## 3. Sample Preparation and Experimental Intervention

*Material procurement*: All the required materials involved directly or indirectly in the experiment were procured from various sources and are listed in Table 1 along with their respective characteristics, composition, and specifications for clinical use.

**Table 1:** List of materials, manufacturers, and specifications.

Materials	Trade Name/Manufacturer	Specifications/Features
Tetric N-Ceram Code P	Ivoclar Vivadent AG Bendererstrasse 2 FL-9494 Schaan Liechtenstein	Lot number: Z05VRM (Shade A2) Light-cure, packable (bulk filled radiopaque nanohybrid composite), nano-optimized filler (16 shades) Resin matrix—Bis-GMA, Bis-EMA, and urethane dimethacrylate monomer (UDMA), light initiator Ivocerin; fillers—barium aluminum silicate glass (two particle sizes), filler content 61% (volume), and 17% polymer fillers or “Isofiller” Increment—4 mm, curing time 10 s (>1000 mW/cm^2^). Cylinder-shaped specimens—5 mm diameter × 3 mm height
Tetric N-Flow Shade A2 Filling material Code F	Ivoclar Vivadent AG Bendererstrasse 2 FL-9494 Schaan Liechtenstein	Lot number: Z05J90 (Shade A2) Light-curing, radiopaque flowable nanohybrid composite (according to ISO 4049 [58] Type 1, Class 2, Group 1). Composition: barium glass, Bis-GMA, UDMA, ytterbium trifluoride, TEGDMA, barium–aluminum fluorosilicate glass, Si-Zr mixed oxide [inorganic fillers: 38–40 vol%, particle size: 0.03 μm and 15.5 μm] Available in 10 shades (6 enamel, 1 dentin, 1 incisal, and 2 bleach shades) Curing time 10 s (light intensity of 1000–1300 mW/cm^2^), 20 s (500–900 mW/cm^2^); wavelength range of 400–500 nm
Assure Plus bonding resin	Reliance Orthodontic Products, West Thorndale Ave. Itasca, IL, USA	Lot number: 2,310,284 All Surface Bonding Resin (light cure) Bis-GMA, ethanol, MDP, HEMA Application time = 10 s, drying time = 60 s, curing time = 10 s
Transbond XT Light cure adhesive paste	3M Unitek, South Peck Road Monrovia, Los Angeles, CA, USA	Lot number: 9,954,484 Light cure adhesive paste Silane-treated quartz (70–80% in weight), bisphenol A diglycidyl ether dimethacrylate, bisphenol A bis(2-hydroxyethyl ether) dimethacrylate, silane-treated silica, diphenyliodonium
Ceramic bracket Code C	Ortho organizer, San Marcos, CA, USA	Polycarbonate ceramic bracket (polycrystalline) Lower bicuspid universal Surface area = 3.5/3.25
Resin (polyurethane) bracket Code R	Orthoflex, Ortho Technology, Carlsbad, CA, USA	Medical-grade polyurethane for superior strength Dove tail grooves with “Micro Rock” coating provide superior mechanical lock (provides millions of additional undercuts to grip the adhesive) No plastic primers needed for bonding Lower bicuspid universal: surface area = 3.9/3.36
Thermocycling machine	Model 1100, SD Mechatronik, Bayern, Germany	Alternate immersion in warm followed by cold liquid simulates high temperature changes Warm bath temperature: 25 °C to 55 °C Cold bath temperature: 5 °C to 15 °C Exposure time—adjustable per bath from 0 to 999 s
Teflon mold	Guarniflon Spa, Castelli Calepio, BG, Italy	Polytetrafluoroethylene sheet 100 mm/100 mm sheet Temperature range −200 °C to 260 °C

*Standardizing specimens*: A medical-grade polytetrafluoroethylene (Guarniflon Spa, Castelli Calepio BG, Italy) sheet was first heat-pressed around a disk-shaped metal die with a 10 mm diameter and 4 mm thickness to yield a Teflon mold that would be used to cure the composite resin specimens. The packable composite (Tetric N-Ceram, Ivoclar Vivadent AG, Schaan, Liechtenstein) was then placed into the Teflon mold in increments of 3 mm, followed by light curing (LED, X-Cure, Woodpecker, Guilin, China). The final uncured layer was compressed against a glass slide after covering it with a celluloid strip followed by the application of pressure. For specimens of flowable composite (Tetric N-Flow, Ivoclar Vivadent AG, Schaan, Liechtenstein), the material was flowed directly into the mold in increments of 2 mm followed by curing and then filling the remaining mold. A 2 mm layer thickness was standardized by marking 2 mm depth on the outside of the transparent mold. Table 1 presents the curing specifications for both composites.

*Specimen grouping* (Figure 1): A total of 96 composite resin disks [48 flowable, 48 packable] were fabricated, which at this stage were divided into two main groups, aged (A) and non-aged (N). A total of 24 specimens of flowable (F) and an equal number of packable (P) composites served as controls (non-aged), while the other 48 specimens [24 flowable, 24 packable] served as test specimens. For each composite type (flowable and packable), 12 specimens were bonded with ceramic brackets and 12 with resin brackets, thus yielding a total of eight subgroups [control = Gp NFC, Gp NFR, Gp NPC, Gp NPR; experimental = Gp AFC, Gp AFR, Gp APC, Gp APR] with symbol representations as follows: N = non-aged, A = aged, F = flowable composite, P = packable composite, C = ceramic bracket, R = resin bracket; for example, NFC = non-aged flowable composite bonded to ceramic bracket. Both ceramic and plastic brackets were bonded using a standard clinical protocol for orthodontic bracket bonding [9,12,15,17].

*Aging protocol*: The specimens of each composite type were then aged in an artificial accelerated aging procedure using a thermocycling machine (SD Mechatronik, Germany) for 10,000 cycles (5 to 55 degrees centigrade), which represented a total of 12 months of clinical usage [59]. Once the thermocycling was over, all specimens were stored in distilled water at room temperature for a period of 24 h. The specimens were then left to dry at room temperature before the specimens were attached and embedded within a plastic ring using acrylic resin (self-cure). Once the resin was set, the ring carried the specimen with the top smooth surface exposed for bonding the OBs. Care was taken not to contaminate the potential bonding surface with any instrument or finger touch. All samples were handled by a single operator to maintain internal consistency.

## 4. Orthodontic Bracket Bonding

A second mandibular premolar bracket was chosen for both materials. The surface area of both bracket types was first measured using a digital caliper, with the surface area of the plastic brackets (3.9 by 3.36 mm^2^) being more than that of the ceramic brackets (3.5 by 3.25 mm^2^).

*Surface treatment*: Standard mechanical surface treatment employed for all specimens in all subgroups included the sandblasting [Al_2_O_3_ (aluminum trioxide, Korox 50, Lincoln, RI, USA); size = 50 μm] using an intraoral sandblasting device (Airsonic mini sandblaster, Hager & Werken, Germany) with settings mimicking clinical use [2.5 bars pressure, 10 mm away from the sample, duration of 30 s] with the point of the machine vertical to the sample surface. Each specimen was then prepared for chemical surface treatment, which was accomplished by applying a single coat of silane bonding agent [Assure Plus, Reliance Orthodontic Products, West Thorndale, USA] using a micro brush, followed by drying for 60 s and light curing of 10 s as per the manufacturer’s instructions. The adhesive paste Transbond XT (light cure) (3M Unitek, South Peck Road Monrovia, Los Angeles, CA, USA) was then uniformly applied to the base of each orthodontic bracket [ceramic and plastic] and centered onto each composite resin specimen using a scaler tip (KaVo Perio Tip number 8, length: 38 mm, weight: 1.2 g). A load of 200 g was applied onto the center of each bracket for 10 s by using a surveyor. The assembly was then light cured with an LED curing light (Ortholux Luminous; 3M Unitek; output: 1600 mW/cm^2^). The light cure tip was positioned consistently (10 mm away) and at a constant interval (24 s to 12 s on two preset sides) for all specimens. For all specimens, a single operator carried out the bonding technique. Extra unset resin was first removed by the same scaler tip that was used to place the OB in place.

## 5. Measures, Data Collection, and Data Analysis

**Figure 2:**
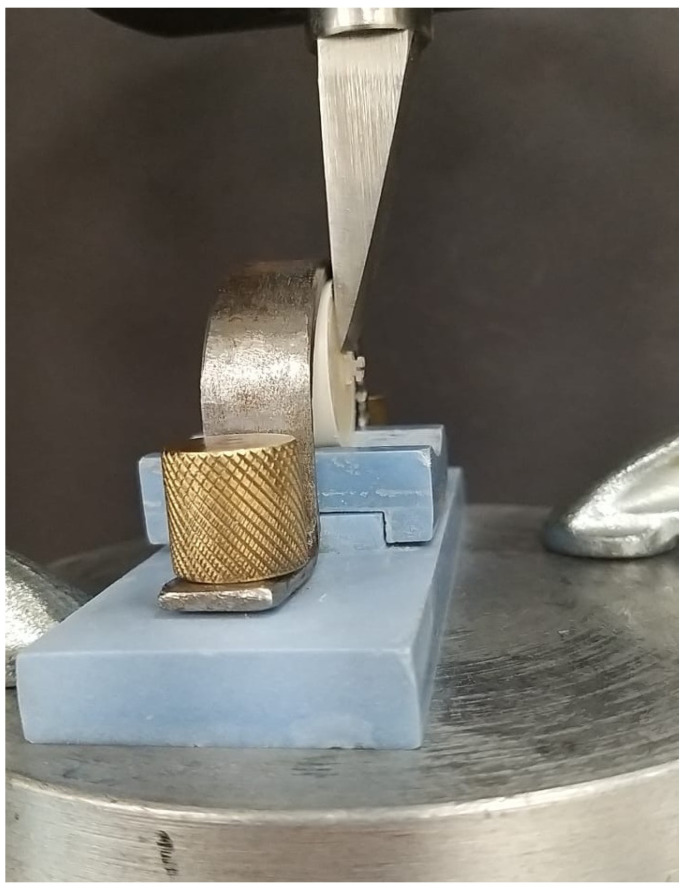
A sample was mounted on the universal testing machine and subjected to a shear bond strength test.

*Specimen clamping* (Figure 2): The clamp (lower jaw) of a universal testing machine (Instron 5965, Instron Corporation, Norwood, MA, USA) was used to secure each specimen in order to assess its bond strength. The specimens were fastened, and then the flat-ended debonding steel rod was moved at a crosshead speed of 1 mm per minute to point at the intersection of the bracket and the specimen (specimen base parallel to the force direction) till the OB failed and the reading at which it failed was automatically generated on the digital display.

*SBS*: The machine automatically generated the load that caused the OB to debond from the underlying composite resin specimen on its display (Figure 2). The load generated was depicted in newtons, which was converted to megapascals using the mathematical formula SBS (Mpa) = failure load (newtons)/surface area (mm^2^) of the OB (σ = F/A).

*Adhesive remnant index (ARI)*: Every surface of the debonded specimen was inspected using a digital microscope with a 20× magnification (KH-7700, Hirox, Tokyo, Japan). The ARI assesses the specimen’s bracket failure by scoring (0 = no residual adhesive, 1 = less than 50% of leftover adhesive, 2 = more than 50% of leftover adhesive, and 3 = all glue left over with distinct bracket mesh marks). Under a microscope, three types of failure were identified: cohesive failure, which happened inside the adhesive; adhesive failure, which was debonding at the adhesive/substrate interface; and mixed failure, which was a combination of these two or a partial adhesive present with either the substrate or the bracket.

*Statistical analysis*: A Microsoft Excel sheet was used to enter raw data, which were subsequently refined, rectified, and coded after visualization. A laptop computer [Lenovo, Windows 11 Pro] was used to run the statistical package for social sciences software SPSS (version 24, IBM, Armonk, NY, USA) and load the coded data for data analysis. Before testing for group variance homogeneity, each subgroup’s data underwent a normalcy test (Shapiro–Wilk) to examine the data distribution. A one-way analysis of variance (ANOVA) test was used to find out how the mean SBS values of each subgroup related to each other independently. The different subgroups were analyzed for differences in the sample averages using the Tukey HSD (honestly significant difference) post hoc pairwise comparison test. If the difference was less than or equal to 0.05 (*p* ≤ 0.05), the probability ‘*p*’ value was deemed significant for statistical analysis. The mean SBS for each experimental (aged) group was tested against its compatriot control (non-aged) group, while they were also compared against the mean values obtained in other subgroups to identify a general indicator for material. The ARI scores were descriptively expressed as frequency percentages in terms of their mode of failure.

## 6. Results

*Data distribution (normality)*: Before running inferential tests, the data related to the SBS of each subgroup were subjected to normality tests so that appropriate inferential tests would be employed. For all individual subgroups, the mean values for SBS showed that the data were normally distributed.

*SBS (Mpa)*: Table 2 presents the mean SBS values obtained for each group and the comparative differences between various subgroups based on aging, type of OB used, and type of restorative surface. Irrespective of the OB type used or the composite resin surface, the samples in the non-aged group, or controls, showed SBS values higher than the samples in the aged group, indicating that aging of the composite resin is associated with decreased SBS. This finding therefore implicates that a clinician must establish the age of the composite restoration from the patient’s treatment history and decide accordingly whether the old composite restoration should be replaced. Such replacement restorations besides affecting treatment outcome also implicate additional treatment cost to the patient. Among both non-aged and aged composite groups, the highest SBS was obtained when ceramic brackets were bonded to flowable composite (NA = 8.19 MPa, A = 7.73 MPa) as compared to packable (NA = 6.46, A = 6.34 MPa). Resin brackets also showed higher bond strength with flowable composites than packable composites for both non-aged and aged samples. This indicates that flowable composites had a better SBS with flowable composites for both ceramic and resin brackets. Generally for a large restoration, bulk fill or packable composites are indicated as they allow incremental bonding, thereby minimizing polymerization shrinkage of the completed restorations. On the other hand, flowable composites are indicated as preventive resin restorations and small class I, II, III, and V restorations. Since OBs are bonded to the labial/buccal tooth surfaces where only class V aged composite restorations are encountered, old packable composite restorations may be replaced with new flowable composite restoration to ensure a durable OB bond for long-term treatments. Between ceramic and resin brackets, the ceramic brackets resulted in higher bond strengths in both non-aged and aged samples for both flowable [Gp NFC = 8.196, AFC = 7.73 MPa] and packable [Gp NPC = 6.469, APC = 6.348 MPa] composites. Resin OB when bonded with aged packable composite showed the least SBS in both non-aged [5.141 MPa] and aged [4.895 MPa] subgroups, indicating that resin OB show a weak SBS with packable composites. These findings reiterate the superiority of ceramic brackets over resin-based polyurethane brackets in terms of providing a durable bond. While plastic brackets are cheaper options for patients, a clinician must ensure that their treatment goals are not compromised by providing cheap alternatives to the patient treatment. The one-way ANOVA test on the mean values of SBS among all subgroups was observed to be statistically significant at the probable ‘*p*’ value of 0.05. The clinical threshold of minimum SBS between 6 and 8 MPa was not observed among groups that used resin brackets irrespective of aging or bonding with packable and flowable composites, thus indicating that resin brackets do not produce adequate bond strength against composite surfaces.

The differences in mean SBS and the significance of these differences for each subgroup against its respective control and the means obtained for other subgroups [non-aged and aged, ceramic and resin OB, flowable and packable composites] were obtained through the post hoc Tukey HSD test and are presented in Table 3. When compared against respective controls, all subgroups showed a reduction in SBS between non-aged and aged in both flowable and packable groups; however, these differences were not significant, thus indicating that aging of the specimens did not show a significant reduction in bond strengths in both composite types using both types of OBs. In addition, the SBS of flowable composites did not differ significantly between Gp NFR and Gp NPC, Gp NFR and Gp APC, Gp AFR and Gp NPR, and Gp AFR and Gp APR (Table 3). The mean SBS of packable composites did not differ significantly between Gp NPC and APC, Gp NPR and Gp APC, Gp NFR and Gp AFR, and Gp APR and Gp NPR. These findings indicate that the SBS in these combinations of aging, OB type, and composite material type were almost similar, although some of them were below the clinical threshold of 6 to 8 MPa.

**Figure 3:**
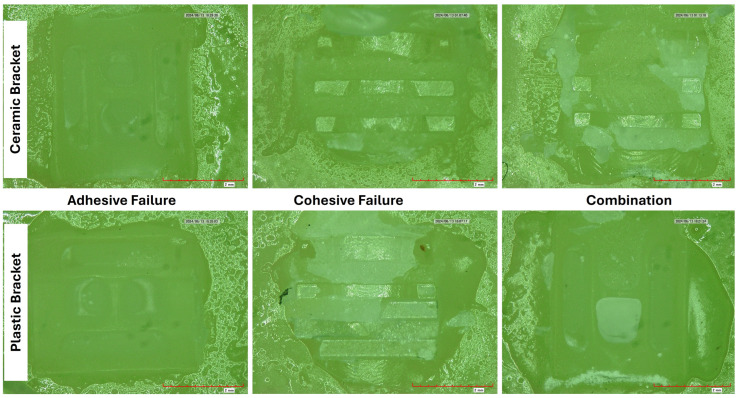
Microscopic exemplary images of the different types of failures observed (adhesive, cohesive, and combination) using ceramic and plastic brackets showing the amount of adhesive left after shear bond strength testing.

*Adhesive remnant index scores (ARI)* (Figure 3): The frequency distributions of ARI scores [0–3] and their respective failure modes [cohesive, adhesive, mixed] obtained in different subgroups based on aging, composite, and OB types are presented in Table 4. All ceramic bracket groups, irrespective of aging and composite type, showed higher frequencies of scores 1 and 2 on ARI, indicating that the adhesive was left on the specimen surface. Removing the adhesive from the surface can be time consuming besides being annoying and cumbersome to the patient’s ability to maintain oral hygiene. A clean polished surface is essential to ensure self-cleansing. The ceramic OBs also showed a higher frequency of cohesive failures except in Gp NPC, where the mixed type of failure was highest (50%) (Figure 3). On the contrary, all subgroups using plastic OBs showed a higher percentage of adhesive failures, with Gp AFR and Gp APR showing 100 percent of the adhesive failures. These results indicate that with plastic brackets, less adhesive is left over the aged composite surface while a higher amount of adhesive is left when ceramic OBs are used.

## 7. Discussion

This in vitro study evaluated the SBS between aged flowable and packable composite resin restoration material and two different aesthetic brackets, ceramic and plastic. The significant findings of the study include that aged composites (up to 1 year) irrespective of type show decreased SBS to OBs irrespective of their type and therefore should be replaced whenever encountered in orthodontic practice. However, flowable composites showed clinically acceptable bond strength even after aging; therefore, they may be used to bond OBs if they are old and ceramic brackets are used. For plastic brackets, the bond strengths against non-aged and aged were lower than clinically acceptable thresholds; therefore, they need to undergo further refinement in terms of their designs. Air abrasion as a means of surface treatment may not be sufficient enough to produce adequate bracket bond strength; therefore, additional surface treatments of aged composites need further investigation. The bond strength for non-aged composites in this study ranged from 5.14 MPa for resin brackets to 8.19 MPa for ceramic brackets. Della Bona A et al. [28] reported SBS of ceramic brackets to aged resin composite in the range of 10 to 13 MPa, which is higher than our study. The differences in the results are due to different aging processes (37 °C deionized water for 23 days) and the use of different surface treatments (acid etch, 38% phosphoric acid, diamond bur) as compared to ours, which utilized 10,000 cycles between 5 and 55 degrees and air abrasion. A 10,000 thermocycling was chosen because the changes in the bond strength that can occur due to thermal expansion coefficients and water sorption can be observed only when thermal cycling is performed for long time periods. Contrarily, studies that have utilized 1000 cycles do not represent adequate clinical time, nor do they reflect the changes in the bond due to thermal coefficient changes and water sorption by the resin restorations. Bayram M et al. [1] reported an SBS of 10.29 MPa after 1000 cycles while using metal brackets with aged composites. His study compared multiple surface treatments [38% phosphoric acid, 9.6% hydrofluoric acid, airborne aluminum trioxide, sodium bicarbonate particle, and diamond bur] and two different aging protocols [first water storage for 7 days (37 °C) followed by thermocycling [(1000 cycles) (5–55 °C)]. The SBS values for ceramic brackets to aged and non-aged composites have varied among different studies. Blakey M et al. [7] found very low SBS [2.87 MPa] for ceramic brackets bonded to provisional polycarbonate crowns after various surface treatments. Eslamian L et al. [29] had SBS values ranging between 12.85 MPa for metal brackets and 26.68 MPa for ceramic brackets to non-aged resin composites after different surface treatments [HF (5%), air abrasion (50 μm alumina), and diamond bur]. However, their aging was performed after the OBs were bonded and was only 500 cycles, which explains the higher SBS values. The values obtained in their study are comparable to those obtained when ceramic brackets are bonded to natural teeth (Ansari MY et al. 23.4 to 27.26 MPa) [60]. The differences in SBS values are explainable on the basis of the aging technique (before and after OB bonding). In vitro composite aging investigations have used thermocycling and storage in aqueous solutions or citric acid [42,61]. The physical properties of composite materials can be altered by the constant exposure to ultraviolet (UV), visible (VIS), and distilled water that occurs during accelerated aging [62]. Structural/chemical characteristics and surface treatment influences differences that also exist between aged and fresh composite resins. An aged resin-based composite’s surface treatment removes the saliva-altered superficial layer to reveal a fresher, higher-energy composite surface and increases surface area with surface imperfections. Traditional and modern resin-based composites can be bonded in three ways: chemically to the organic matrix, chemically to the exposed filler particles, and micromechanically to the treated surface [63]. Unconverted C=C double bonds on the aged composite surface are responsible for resin matrix bonding. Whether they can considerably enhance binding strength using a wetting agent (bonding system) needs more investigation [63]. Chemical bonds to the matrix, chemical bonds to the exposed filler particles, and micromechanical retention caused by monomer components penetrating matrix microcracks are the three mechanisms of bracket bonding with intermediate adhesive [64]. Composite layers are therapeutically attached by an oxygen-inhibited layer of non-polymerized resin [65]. However, old restorations do not have the unpolymerized layer on top. Adhesion compared to a newly made composite decreases with time due to a decrease in the amount of unreactive methacrylate groups found in intermediate adhesive agents [63]. Thus, bracket bonding depends on restoration age. The increased ceramic bracket SBS in our study is related to its better ability to transmit curing light, which reflects in better and efficient photopolymerization [4,16,24]. Another factor is homogenous mechanical irregularities caused by air abrasion, which has been reported with higher bond strength irrespective of substrate, bracket type, and bonding agent.

Our results show that polyurethane-based resin brackets showed comparatively lower SBS values across all groups as compared to ceramic brackets. The values obtained in our study are less than those obtained in a previous study, which reported a range of 8 to 14 MPa [45]. However, the brackets used in their study had a mechanical slot, and the resin was not aged. Study results on the SBS of plastic brackets to composite resin suggest that plastic brackets need to be investigated for other means of providing retention. These may include new bracket anatomical base designs that can include serrations, grooves, or small plastic pins incorporated within the bracket base. Surface treatment of the restoration, both mechanical and chemical, also needs to be further investigated to identify the best possible surface treatment of composite resin that would enhance the SBS of the plastic bracket. Since there are comparatively no studies that have investigated the SBS of ceramic and resin OBs to aged resin, a comparison with the use of a stainless steel bracket system with aged composite would give a better understanding of the results of this study. The range of SBS values obtained in our study falls in complete agreement with earlier studies conducted using stainless steel OBs and aged resin. Using sandblasting, diamond bur, and three primers, Tayebi A. et al. investigated how surface preparation affected the SBS (SBS) of metal brackets to bulk-filled aged composite [2000 thermal cycles]. High SBS between brackets and aged composite surfaces was adequately achieved by all primer and surface preparation combinations, according to the results. SBS for diamond bur was between 7.57 and 10.8 MPa, and for sandblasting, it was between 9.94 and 13.8 MPa [34]. Seyhan-Cezairli N et al. [55] in his study on SBS of metal brackets with aged bulk fill composites [500 cycles] and single surface treatment had a range of SBS values between 2.55 and 9.29 MPa, with non-aged having higher SBS than aged composites. Comparatively, our SBS values for packable ranged between 4.89 and 6.46 MPa, which is lower; however, the differences are mainly due to differences in aging [500 cycles vs. 10,000 cycles]. Danha LS et al. [27] used flowable composite as a bonding agent as a medium to bond sapphire brackets to composite restoration and found the SBS to be lower than the Transbond bonding adhesive paste. Farhadifard H et al. [54] assessed the SBS of ceramic brackets to old nanohybrid composite restoration using different surface treatments [acid etching, sandblasting, grinding, and Er, Cr:YSGG laser irradiation], with bur grinding showing the highest SBS values (9.16 MPa) and sandblasting (8.13 MPa). The bonding methods of ceramic brackets can be classified as mechanically retentive bases, chemically retentive bases coated with silane, or a combination of the two, and they provide benefits such as biocompatibility, aesthetics, resistance to temperature and chemicals, and superior bond strength [66]. Numerous investigations have revealed that silane-treated chemically retentive brackets have a stronger binding between ceramic and composite than mechanically retentive brackets [22,23,24,25,26,50,54,60]. This strong bond strength practically matches enamel strength, making enamel fractures further frequent. So, ceramic bracket bases that are mechanically retentive are superior to chemically retentive ones [60]. For mechanical retention, manufacturers provide a variety of base designs, such as microcrystalline, polymeric, dimpled, mechanical, dovetail, and mechanical ball. Some claim that the bond strengths and debonding of traditional stainless steel brackets, which use a mesh welded to the bracket base for mechanical retention, are not uniform [60]. There are two methods for achieving bonding: using a precoated bracket system or adding adhesive to the base of the bracket by hand before placing it [22,23,24]. The flowable composite bonding process uses glycerol phosphate dimethacrylate (GPDM) adhesive monomer, which bonds with calcium ions of the tooth substrate through acid etching and chemical bonding [21]. The material gains mechanical strength through cross-linking methacrylate functional groups with additional monomers. The increased binding strength of flowable composites compared to the packable composites may also be due to polymerization kinetics and contraction stress dynamics [27,67]. When using an adhesive with a resin composite filled with more fillers, the polymerisation shrinkage stress of the resin composite will strain the bond produced by the adhesive on the dental substrate. The flowable composite is projected to convey less contraction stress to interfaces during curing due to its low elastic modulus [21,27]. The storage media has been reported to influence the properties of unfilled resins in general [68].

Our results on the ARI scores show that ceramic brackets left more adhesive on the surface for score 3 (more than 50%) while plastic brackets did not have any score of 3 for all subgroups. The amount of adhesive left over the surface indicates two significant clinical applications, one being that the surface of the substrate (in this case composite) will not undergo deformation or fracture (like tooth enamel), and secondly it means that the clinician has to spend more time in removing adhesive from the surface, which is time-consuming and cumbersome. The frequency distribution of score 0 (no adhesive left) was also higher in plastic brackets than ceramic brackets. The interpretation of these findings is similar to those observed in a study by Ansari MY et al. [60] and Sibi AS et al. [69] in relation to ceramic brackets and Ali O et al. [45] and Guan G et al. [47] for plastic brackets, despite there being differences in the studies regarding the substrate. Debonded brackets with a lower ARI score of 1 had a relatively weaker bond strength because there was more adhesive on the bracket base and less on the tooth surface. Surface roughness, surface texture, the shape of the bracket surface (base), and the surface chemistry between the material and the adhesive are some of the factors that can affect ARI scores [34,70].

*Strength and limitations of the study*: The main strength of the study is its novelty in investigating plastic brackets to aged composite restorations that have not been investigated as yet. Despite the strength, the study is limited because of being in vitro, which cannot completely replicate the oral conditions, the brands used for brackets and the composites are not all, and the effects of other surface treatments have not been investigated. Irrespective of in vitro study designs, various factors that are present in the oral cavity cannot be accurately simulated in the laboratory, which include the changing pH of the saliva and the oral cavity that change due to systemic conditions and the diet of the patient. Similarly, the salivary composition and amount vary from individual to individual and within an individual at different times of the day. Lack of simulation of these oral conditions therefore limits the findings of our study.

*Clinical relevance*: The SBS with aged flowable and packable composites is clinically nonacceptable with plastic brackets, but it is acceptable with ceramic brackets. More glue is left on the composite surface by ceramic brackets, making cleaning them more of a challenge.

## 8. Conclusions

The study, within its scope, aims, and limitations, concludes that the SBS of ceramic and plastic OBs is comparatively lower when bonded against aged flowable/packable composites using air abrasion as surface treatment. Aged flowable composites had clinically acceptable SBS when ceramic brackets were used, while aged packable had lesser SBS values when ceramic and plastic brackets were bonded. The bond strength of flowable composites when aged was more than that of packable composites. Ceramic brackets had higher bond strength than plastic brackets irrespective of composite type. The ARI scores obtained show more cohesive failures when ceramic brackets were used while more adhesive failures occurred when plastic brackets were used. With ceramic brackets, more adhesive was left over the surface, indicating more clinical time was required to remove it.

## Figures and Tables

**Table 2:** Comparative differences in means of shear bond strength (Mpa) between orthodontic brackets (ceramic and polyurethane) to composite resin (flowable, packable) before and after aging (10,000 cycles or 12 months clinical use).

Independent Variable	Composite Type	Bracket Type	n	Subgroup	Mean	SD	dF	F Statistic	*p*-Value
Control Groups Non-Aged (Code N) (n = 48)	Flowable (Code F) (Tetric N-Flow) (n = 24)	Ceramic (Polycrystalline) (Code C)	12	NFC	8.196	0.544	7	35.502	0.0000 *
		Resin (Polyurethane) (Code R)	12	NFR	5.838	0.684			
	Packable (Code P) (Tetric N-Ceram) (n = 24)	Ceramic (Polycrystalline) (Code C)	12	NPC	6.469	0.645			
		Resin (Polyurethane) (Code R)	12	NPR	5.141	0.801			
Experimental Groups Aged (Code A) (n = 48)	Flowable (Code F) (Tetric N-Flow) (n = 24)	Ceramic (Polycrystalline) (Code C)	12	AFC	7.73	0.545			
		Resin (Polyurethane) (Code R)	12	AFR	5.558	0.694			
	Packable (Code P) (Tetric N-Ceram) (n = 24)	Ceramic (Polycrystalline) (Code C)	12	APC	6.348	0.776			
		Resin (Polyurethane) (Code R)	12	APR	4.895	0.746			

Abbreviations: SD = standard deviation; group description/coding: N = non-aged, A = aged, F = flowable composite, P = packable composite, C = ceramic bracket, R = resin bracket, example NFC = non-aged flowable composite ceramic bracket. Test employed; one-way analysis of variance (ANOVA) for differences within groups and Tukey HSD (honestly significant difference). Statistical significance: All differences at various time intervals in each group were considered to be statistically significant if the probable ‘*p*’ value was ≤0.05. “*” indicates statistical significance.

**Table 3:** Tukey HSD (honestly significant difference) post hoc pairwise comparison showing differences in the mean shear bond strength s within subgroups based on aging (aged/non-aged), composite resin type (flowable/packable) and bracket type (ceramic/polyurethane resin).

Subgroups	NFC	AFC	NPC	APC	NFR	NPR	AFR	APR
NFC		0.47	1.73	1.85	2.36	2.64	3.06	3.3
		0.708	0.0000 *	0.0000 *	0.0000 *	0.0000 *	0.0000 *	0.0000 *
AFC	0.47		1.26	1.38	1.89	2.17	2.59	2.83
	0.708		0.0005 *	0.0000 *	0.0000 *	0.0000 *	0.0000 *	0.0000 *
NPC	1.73	1.26		0.120	0.630	0.910	1.33	1.57
	0.0000 *	0.0005 *		0.9999	0.3321	0.0332 *	0.0002 *	0.0000 *
APC	1.85	1.38	0.120		0.51	0.79	1.21	1.45
	0.0000 *	0.0000 *	0.9999		0.609	0.103	0.0010 *	0.0000 *
NFR	2.36	1.89	0.630	0.51		0.28	0.70	0.94
	0.0000 *	0.0000 *	0.3321	0.609		0.973	0.214	0.023 *
NPR	2.64	2.17	0.910	0.79	0.28		0.42	0.66
	0.0000 *	0.0000 *	0.0332 *	0.103	0.973		0.812	0.270
AFR	3.06	2.59	1.33	1.21	0.70	0.42		0.25
	0.0000 *	0.0000 *	0.0002 *	0.0010 *	0.214	0.812		0.987
APR	3.3	2.83	1.57	1.45	0.94	0.66	0.25	
	0.0000 *	0.0000 *	0.0000 *	0.0000 *	0.023 *	0.270	0.987	

Note: Group description/coding: N = non-aged, A = aged, F = flowable composite, P = packable composite, C = ceramic bracket, R = resin bracket, example NFC = non-aged flowable composite bonded to ceramic bracket. Test employed; one way analysis of variance (ANOVA) for differences within groups and Tukey HSD (honestly significant difference). Statistical significance: All differences at various time intervals in each group were considered to be statistically significant if the probable ‘*p*’ value was ≤0.05. Statistical significance: All differences between various subgroups were considered to be statistically significant if the probable *p* value was ≤0.05. “*” indicates statistical significance.

**Table 4:** Frequency distribution of adhesive remnant index and mode of failures for various subgroups based on aging (aged/non-aged), composite resin type (flowable/packable), and bracket type (ceramic/polyurethane resin).

		ARI Score Distributions				Failure Mode Distribution		
Subgroups		0	1	2	3	C	A	M
NFC	N	1	5	5	1	9	0	3
	%	8.33%	41.67%	41.67%	8.33%	75.00%	0.00%	25.00%
AFC	N	0	7	5	0	8	0	4
	%	0.00%	58.33%	41.67%	0.00%	66.67%	0.00%	33.33%
NPC	N	1	2	7	2	5	1	6
	%	8.33%	16.67%	58.33%	16.67%	41.67%	8.33%	50.00%
APC	N	1	8	3	0	10	2	0
	%	8.33%	66.67%	25.00%	0.00%	83.33%	16.67%	0.00%
NFR	N	3	4	5	0	1	10	1
	%	25.00%	33.33%	41.67%	0.00%	8.33%	83.34%	8.33%
NPR	N	2	3	7	0	1	10	1
	%	16.67%	25.00%	58.33%	0.00%	8.33%	83.34%	8.33%
AFR	N	3	6	3	0	0	12	0
	%	25.00%	50.00%	25.00%	0.00%	0.00%	100.00%	0.00%
APR	N	3	5	4	0	0	12	0
	%	25.00%	41.67%	33.33%	0.00%	0.00%	100.00%	0.00%

Abbreviations: Group description/coding: N = non-aged, A = aged, F= flowable composite, P = packable composite, C = ceramic bracket, R = resin bracket, example NFC = non-aged flowable composite bonded to ceramic bracket. Adhesive remnant index (ARI) scoring criteria: score 0: no adhesive remained on the restoration surface; score 1: less than 50% of the adhesive remained on the restoration surface; score 2: more than 50% of the adhesive remained on the restoration surface; score 3: all the adhesive remained on the restoration surface; the more the adhesive left on the surface, the less was the risk of enamel fracture. Modes of failure: C = cohesive, A = adhesive, M = mixed.

## Data Availability

All relevant data have been presented within the article; however, the raw data files are available from the corresponding author and can be available upon reasonable request.

## Abbreviations

OB—orthodontic brackets; SBS—shear bond strength; A—aged composite; NA—non-aged; F—flowable composite; P—packable composite; C—ceramic brackets; P—plastic brackets; ARI—adhesive remnant index; ANOVA—one way analysis of variance; HSD—honestly significant differences; Mpa—mega pascal; Al_2_O_3_—aluminum trioxide; LED—light emitting diode.
